# The Untapped Biomarker Potential of MicroRNAs for Health Risk–Benefit Analysis of Vaping vs. Smoking

**DOI:** 10.3390/cells13161330

**Published:** 2024-08-10

**Authors:** Ahmad Besaratinia, Stella Tommasi

**Affiliations:** Department of Population & Public Health Sciences, USC Keck School of Medicine, University of Southern California, M/C 9603, Los Angeles, CA 90033, USA; tommasi@med.usc.edu

**Keywords:** epigenetics, gene expression, miRNA, smoking, youth vaping

## Abstract

Despite the popularity of electronic cigarettes (e-cigs) among adolescent never-smokers and adult smokers seeking a less pernicious substitute for tobacco cigarettes, the long-term health impact of vaping is largely unknown. Like cigarette smoke, e-cig vapor contains harmful and potentially harmful compounds, although in fewer numbers and at substantially lower concentrations. Many of the same constituents of e-cig vapor and cigarette smoke induce epigenetic changes that can lead to the dysregulation of disease-related genes. MicroRNAs (MiRNAs) are key regulators of gene expression in health and disease states. Extensive research has shown that miRNAs play a prominent role in the regulation of genes involved in the pathogenesis of smoking-related diseases. However, the use of miRNAs for investigating the disease-causing potential of vaping has not been fully explored. This review article provides an overview of e-cigs as a highly consequential electronic nicotine delivery system, describes trends in e-cig use among adolescents and adults, and discusses the ongoing debate on the public health impact of vaping. Highlighting the significance of miRNAs in cell biology and disease, it summarizes the published and ongoing research on miRNAs in relation to gene regulation and disease pathogenesis in e-cig users and in vitro experimental settings. It identifies gaps in knowledge and priorities for future research while underscoring the need for empirical evidence that can inform the regulation of tobacco products to protect youth and promote public health.

## 1. Introduction

Electronic cigarettes (e-cigs) are a form of electronic nicotine delivery systems (ENDS) that simulate tobacco smoking [1,2,3]. E-cigs have been marketed and advertised originally as safe, and subsequently as a less harmful alternative to combustible tobacco cigarettes [4,5,6]. Since their introduction into the U.S. market in 2007, e-cigs have risen in popularity at a tremendous pace [4,7,8]. In the past few years, e-cigs have become the second most used tobacco product by U.S. adults, with combustible cigarettes topping the list [9]. From 2011 and onward, e-cigs have been massively adopted by teens and youth who have wished to experiment with tobacco products [10]. In 2021, 4.5% of U.S. adults aged 18 and over were current e-cig users, with those between the ages of 18 and 24 having the highest rate of use (11.0%) [11]. Since 2014, e-cigs have been the most commonly used tobacco product by U.S. youth [10]. In 2023, 2.1 million middle and high school students (7.7%) in the U.S. were current e-cig users [12]. Of these, 550,000 (4.6%) were middle schoolers (grades 6–8) and 1.56 million (10.0%) were high schoolers (grades 9–12). Among students who currently used e-cigarettes, the overwhelming majority reported using disposable e-cigs (60.7%) and e-cigs with pre-filled or refillable pods or cartridges (16.1%). Moreover, 89.4% reported using flavored e-cig products, with fruit and candy being the most popular flavors; the top used brands were Elf Bar, Esco Bars, Vuse, JUUL, and Mr. Fog, respectively [12].

E-cig use is colloquially known as “vaping”, and e-cig users are commonly called “vapers” [1,5]. Unlike conventional cigarettes that burn tobacco leaves to generate smoke, e-cigs heat a solution, dubbed e-liquid/e-juice, to produce inhalable vapor [7,13]. Heat-based vaporization of e-liquid produces fewer and generally lower levels of harmful and potentially harmful chemicals than those formed during the combustion of tobacco cigarettes [5,7]. This has led, in part, to the perception that e-cigs are a reduced-harm substitute for tobacco cigarettes [3,4]. However, cumulative exposure of regular e-cig users to the toxicants and carcinogens present in e-cig vapor, irrespective of their quantity and concentrations, makes vaping a definite health risk [7,14]. Elucidating the biological effects of e-cig use can help determine the health risks or potential benefits of vaping relative to smoking. This will, in turn, help resolve a pressing public health problem, concerning millions of adult former smokers and youth never-smokers who have taken up this controversial habit [3,15,16].

The biological effects of many chemical constituents of e-cig vapor and cigarette smoke are attributable to epigenetic alterations that can lead to the dysregulation of disease-related genes [17,18,19,20,21,22,23,24,25,26]. The toxic and carcinogenic compounds present in e-cig vapor and cigarette smoke, such as carbonyl compounds, volatile organic compounds (VOC), free radicals, and heavy metals, can cause a multitude of DNA lesions [7,13], many of which may affect the epigenetic machinery, e.g., by inhibiting the binding of epigenetic enzymes to their targets [24,25,26,27]. There may also exist a competitive demand for the metabolism of e-cig vapor and cigarette smoke-derived compounds [28] and the cofactors/cosubstrates of enzymes required for epigenetic modifications [27]. Furthermore, e-cig vapor and cigarette smoke can impose oxidative stress and induce DNA damage, which may impact transcriptional regulatory elements and other epigenetic marks [23,29,30].

MicroRNAs (miRNAs) play a critical role in the regulation of gene expression in health and disease states [31,32,33,34,35]. As a major class of non-coding RNAs, microRNAs are key regulators of genes involved in the pathogenesis of human diseases, including tobacco-related diseases [36,37,38,39]. Compelling evidence shows the modulatory effects of miRNAs on gene expression in response to specific cues or external stimuli, such as tobacco product use [40,41,42,43,44,45,46]. A large body of research has shown that miRNAs regulate crucial genes involved in the pathogenesis of smoking-related diseases [47,48,49,50,51]. To date, however, the use of miRNAs for investigating the disease-causing potential of vaping has not been fully explored. Elucidating the role of miRNAs in the regulation of disease-related genes in e-cig users has tremendous translational potential. Identifying miRNAs that regulate disease-specific genes in e-cig users can translate to the discovery of biomarkers of exposure and effects for vaping. These biomarkers will be instrumental in determining the health risks or potential benefits of vaping relative to smoking. Scientifically driven risk–benefit analysis of vaping vs. smoking can inform regulatory agencies, including the U.S. Food and Drug Administration (FDA), which has the authority to regulate the manufacturing, marketing, and distribution of tobacco products to protect public health [3,52,53].

This review article provides an overview of e-cigs, trends in use among adolescents and adults, and challenges of research on the safety, efficacy, and health risks or potential benefits of vaping relative to smoking. While highlighting the significance of miRNAs in cell biology and disease, it summarizes the published and ongoing research on miRNAs in relation to gene regulation and disease pathogenesis in e-cig users and in vitro experimental settings. The gaps in knowledge and priorities for future research are discussed, whilst the need for empirical evidence that can inform the regulation of tobacco products to protect youth and promote public health is underscored.

## 2. The Public Health Impact of Vaping

There has been a contentious and ongoing debate over the public health impact of vaping [3,54,55,56,57]. Vaping advocates claim that e-cig use, especially when combined with behavioral counseling, aids in smoking cessation [3,4]. Garnering support for this claim, meta-analyses of randomized clinical trials have shown that e-cigs can help adult smokers quit [15,53]. An important caveat is that e-cigs have been shown to assist smokers quit only when they have been used as a medical intervention in clinical trial settings [4,15,58,59,60,61]. However, numerous population-based studies have documented that e-cigs, as a consumer product, are not effective in smoking cessation [15,53,62,63,64,65]. Currently, nowhere in the world are e-cigs approved as a medical intervention. Instead, e-cigs are consumer products, purchasable by anyone over a certain age, and usable as much or as often as one desires [16,64]. Another claim is that the marked decline in youth smoking in recent years is driven by vaping [4,66]. The counterargument is that vaping has facilitated the experimentation of teens and youth with tobacco products [16,56,67]. Consequently, a new generation of adolescents, youth, and young adults who would have never smoked in the first place has become addicted to nicotine [1,2,52]. It is also argued that vaping may reverse the decades-long successful public health campaign against smoking and “renormalize” this unhealthy habit [60,68,69]. Whilst proponents of harm reduction contend that e-cigs are a viable alternative to the irrefutably dangerous tobacco cigarettes [3,4,54], more evidence emerges on the adverse health consequences of vaping [1,5,7,13].

## 3. Overview of E-Cigs

E-cigs are handheld battery-powered devices that mimic the feel and look of tobacco cigarettes [6,7,55]. E-cigs heat a solution (e-liquid/e-juice) that contains a base solvent (humectant: propylene glycol (PG) and glycerin/vegetable glycerin (VG) at mixed ratios), nicotine, and a wide variety of flavors and additives at different concentrations [5,6,7]. The heating and subsequent vaporization of e-liquid renders an aerosol (“vapor”) that users inhale through a mouthpiece. While the ingredients of e-liquid are generally recognized as safe (GRAS) for ingestion, inhalation of these ingredients cannot be considered risk-free [5,7,13]. Since their introduction into the U.S. market in 2007, e-cigs have evolved rapidly and significantly, from the first-generation “cig-a-likes” that were designed to resemble combustible cigarettes, to the second-generation vape pens, third-generation box mods, and fourth-generation pod-based devices, which are well-liked by teens and youth [1,5,7]. The substantial changes in the features and design of e-cigs have been accompanied by tremendous diversifications of e-liquid [70,71,72]. Numerous chemicals have been added to e-liquid to produce products that appease virtually every user’s desire [13,52,72,73,74]. Currently, there are around 20,000 e-liquids in the market [72,73,75]. The ever-growing number of e-liquids, containing countless combination of chemicals, can expose e-cig users to a wide range of chemicals with known and unknown toxicity profiles [76,77,78,79,80,81,82,83,84].

Many of the same toxic and carcinogenic compounds present in cigarette smoke are also detectable in e-cig liquid and vapor, although in fewer numbers and at generally substantially lower concentrations [1,5,7]. These harmful and potentially harmful chemicals include, but are not limited to, carbonyl compounds, VOC, free radicals, and heavy metals [6,7,13,85]. There are also chemicals that are exclusively detectable in e-cig vapor but not cigarette smoke [73,86]. The latter chemicals likely originate from the mixing of the e-liquid ingredients and/or the vaporization of the base solvent (PG/VG), flavors, or elements leached from the device components [77,87]. There are, at least, seven groups of (potentially) harmful compounds in e-cig liquid and vapor, including carbonyls, VOC, nicotine, nanoparticles, trace metals, bacterial endotoxins, and β-glucans [13]. The lower concentrations of toxicants and carcinogens in e-cig vapor accord with the mode of operation of these devices as e-cigs (unlike traditional cigarettes) do not “burn” tobacco to produce inhalable materials [5,6]. Although the reduced levels of toxic and carcinogenic compounds in e-cig vapor imply a mitigation of health risks, they cannot equate to no risk [84]. It is well established that exposure to many of the same constituents of e-cig vapor at varying concentrations is associated with cardiovascular, immune-related (inflammatory) and respiratory diseases, and cancer, among other diseases [6,7,13,27,28].

## 4. MiRNAs in Health and Disease: Utility for Vaping Research

MicroRNAs (miRNAs) are an evolutionarily conserved class of endogenous single-stranded non-coding small RNAs of approximately 18–24 nucleotides in length [32,88]. MiRNAs are prime regulators of gene expression at the post-transcriptional level [31,89]. MiRNAs exert their regulatory function by binding the 3′-untranslated region (3′-UTR) of messenger RNAs (mRNAs) [90,91]. The base pairing of miRNAs and target mRNAs depends primarily on just seven or eight nucleotides at the 5′ end of the miRNA (called the “seed region”) [37]. An individual miRNA can have multiple mRNA targets, and a single mRNA can be targeted by many miRNAs [31,32,35]. The binding of miRNAs to the complementary sequence of target mRNAs generally leads to mRNA degradation and/or translational repression [33,35,88]. Paradoxically, however, miRNAs can upregulate gene expression by enhancing translation under specific conditions, such as cell cycle arrest [92]. Over 60% of the protein-coding genes in humans are estimated to be regulated by miRNAs [31,89]. While miRNAs are localized intracellularly, they can also be released into the extracellular environment and circulate in various bio-fluids such as plasma, serum, and urine [93,94,95,96,97]. MiRNAs play critical roles in various biological processes, including cell proliferation, differentiation, growth, development, stress response, inflammation, and immunity [88,89,98]. Aberrant expression of miRNAs has been demonstrated in a wide variety of diseases, including cardiovascular disease [47,99,100], respiratory diseases [36,40,48,101,102], immune diseases [49,99,103,104], and cancer [37,38,50,51,105,106,107,108,109], among others [36,39,99]. There is also a mounting recognition of miRNA-mediated dysregulation of gene expression in response to specific cues or external stimuli, such as tobacco product use [40,41,42,43,44,45,46].

There is limited but burgeoning research on miRNA-mediated gene dysregulation in biospecimens from e-cig users [19,110] and in cells treated in vitro with e-cig liquid or vapor [111]. Solleti et al. [111] exposed primary human bronchial epithelial cells differentiated at an air–liquid interface to non-vaporized or vaporized e-cig liquid, with or without nicotine. Exposure of the cells to any e-cig liquid (vaporized or non-vaporized, with or without nicotine) resulted in the induction of oxidative stress-response genes, including glutamate–cysteine ligase catalytic subunit (*GCLC*), glutathione peroxidase 2 (*GPX2*), NAD(P)H quinone dehydrogenase 1 (*NQO1*), and heme oxygenase (decycling) 1 (*HO-1*), as determined using reverse transcription real-time quantitative polymerase chain reaction (qPCR) analysis. Transcriptional profiling of 2541 miRNAs by miRNA-seq analysis identified 578 differentially expressed miRNAs in cells treated with any e-cig liquid (*N* = 12) as compared to untreated controls (*N* = 3). A list of the top 20 differentially expressed miRNAs, including 10 upregulated and 10 downregulated miRNAs, is shown in Table 1. A full list of all 578 differentially expressed miRNAs is available in Supplemental Table S1 in ref. [111]. Additionally, 125 differentially expressed miRNAs were detected in cells treated with vaporized e-liquid (*N* = 6) compared to non-vaporized e-liquid (*N* = 6). Cells treated with nicotine-containing vaporized e-liquid showed the most profound changes in the expression of miRNAs. Several of the differentially expressed miRNAs in e-cig-treated cells were validated using qPCR analysis; these included *MIR26A-2-3P*, *MIR126-5P*, *MIR140-5P*, *MIR29A-5P*, *MIR374A-3P*, and *MIR147B*. Consistent with the overexpression of *MIR126-5P* in treated cells, there was a significant reduction in the expression of its two target genes (i.e., *MYC* and *MRGPRX3*) [111].

Singh et al. [110] analyzed the expression profiles of small RNAs in plasma-derived exosomes obtained from e-cig users (*N* = 7), cigarette smokers (*N* = 7), waterpipe smokers (*N* = 7), dual smokers (both cigarettes and waterpipe) (*N* = 7), and non-users (*N* = 8). RNA-seq analysis showed several RNA biotypes of known biological importance, including Piwi-interacting RNAs (piRNAs), transfer RNAs (tRNAs), small nucleolar RNAs (snoRNAs), small nuclear RNAs (snRNAs), long intergenic non-coding RNAs (lincRNAs), mitochondrial tRNAs (mt_tRNAs), mitochondrial ribosomal RNAs (mt_rRNAs), and miRNAs, in specimens from all groups. MiRNAs comprised 78–81% of all RNA biotypes quantified in various groups. Differentially expressed miRNAs were detected in each group compared to non-users, including e-cig users (seventeen miRNAs, thirteen upregulated, and four downregulated) (see Table 2), cigarette smokers (twenty-four miRNAs, sixteen upregulated, and eight downregulated) (see Supplementary Materials Table S1), waterpipe smokers (sixteen miRNAs, twelve upregulated, and four downregulated) (see Supplementary Materials Table S2), and dual smokers (twenty miRNAs, thirteen upregulated, and seven downregulated) (see Supplementary Materials Table S3).

**Figure 1:**
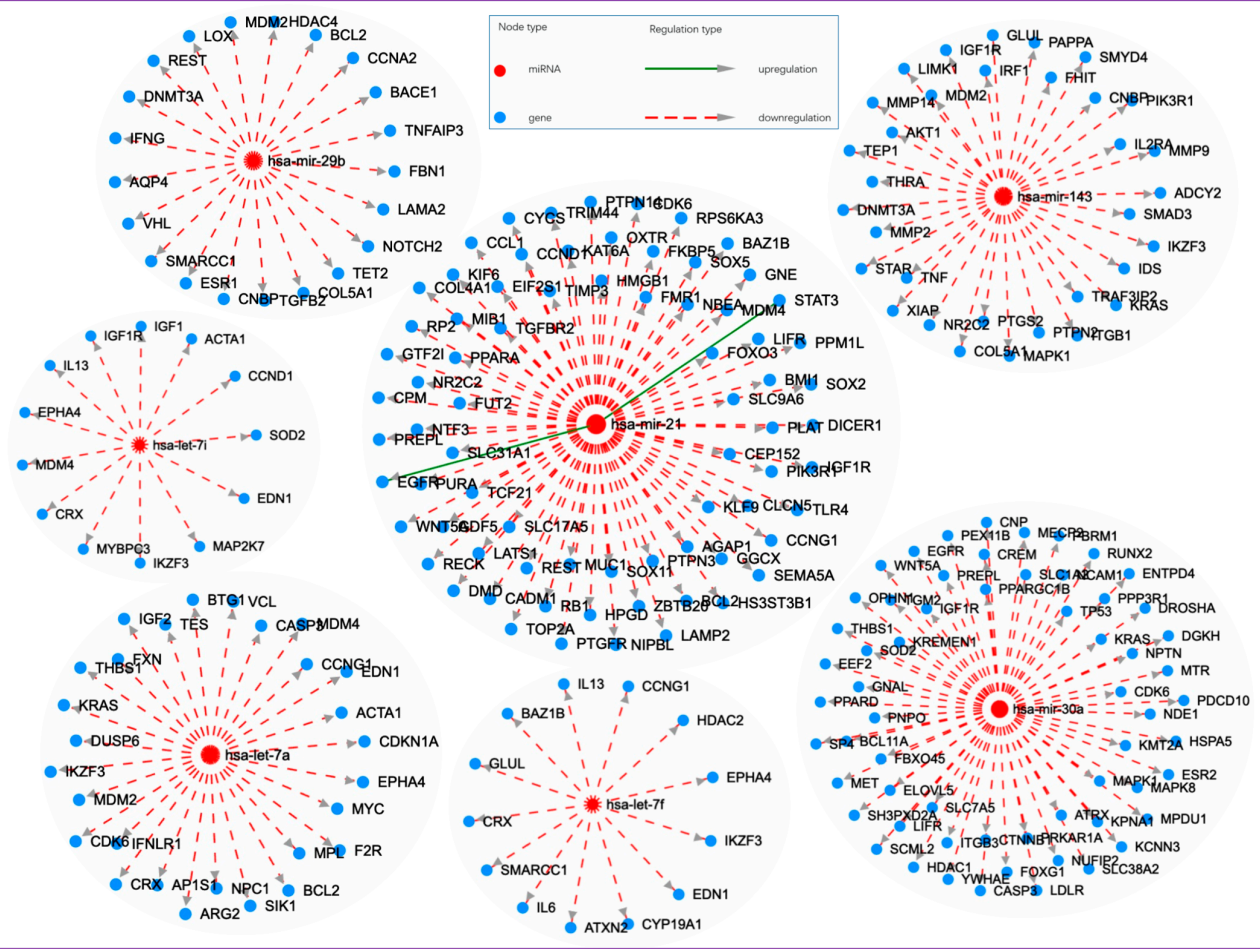
Cytoscape visualization of the networks of miRNA–target interactions (disease–context) for seven dysregulated miRNAs common to e-cig users, cigarette smokers, waterpipe smokers, and dual smokers (both cigarettes and waterpipe) when compared to non-users. The interaction networks are based on the experimentally supported miRNA–target data from miRTarBase (https://mirtarbase.cuhk.edu.cn/ (accessed on 3 March 2024)) using the Human microRNA Disease Database version 4.0 (HMDD v.4.0) (http://www.cuilab.cn/hmdd (accessed on 3 March 2024)). Data are derived from ref. [110].

There were seven overlapping miRNAs common to all four groups when compared to non-users. Figure 1 shows a Cytoscape visualization of the networks of miRNA–target interactions (disease–context) for these seven common miRNAs based on the experimentally supported miRNA–target data from miRTarBase (https://mirtarbase.cuhk.edu.cn/ (accessed on 3 March 2024)) using the Human microRNA Disease Database version 4.0 (HMDD v.4.0) (http://www.cuilab.cn/hmdd (accessed on 3 March 2024)).

**Figure 2:**
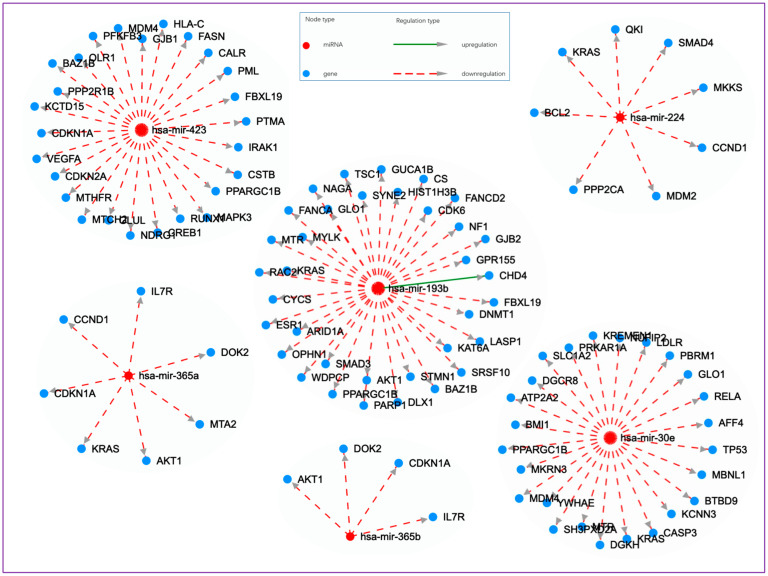
Cytoscape visualization of the networks of miRNA–target interactions (disease–context) for dysregulated miRNAs specific to e-cig users when compared to non-users. The interaction networks are based on the experimentally supported miRNA–target data from miRTarBase (https://mirtarbase.cuhk.edu.cn/ (accessed on 3 March 2024)) using the Human microRNA Disease Database version 4.0 (HMDD v.4.0) (http://www.cuilab.cn/hmdd (accessed on 3 March 2024)). Data are derived from ref. [110].

There were five miRNAs that were specific to e-cig users and not expressed in the other three groups when compared to non-users. Figure 2 visualizes the networks of miRNA–target interactions (disease–context) for these e-cig specific miRNAs using the HMDD v.4.0, as described above. Note that *hsa-miR-365a* and *hsa-miR-365b* are shown separately.

The FunRich gene enrichment analysis of differentially expressed miRNAs identified the most significant functions, including biological pathways, biological processes, molecular functions, cellular components, sites of expression, and transcription factors. The top three biological pathways in all four groups included beta1 integrin cell surface interactions, integrin family cell surface interactions, and TRAIL signaling pathway. The top biological processes were the regulation of nucleobase, nucleoside, nucleotide, and nucleic acid metabolism. The top two molecular functions were related to transcription factor activity and extracellular matrix structural constituents, whereas the top two cellular components were related to the nucleus and cytoplasm. Whilst the top three sites of expression of miRNAs in all four groups were the kidneys, placenta, and skeletal muscle, the lungs were a significant site for miRNA expression in e-cig users, cigarette smokers, and dual users. The top enriched transcription factors in all four groups included EGR1, SP1, SP4, and POU2F1, while ZFP161 was exclusively enriched in e-cig users and dual smokers. The target genes of differentially expressed miRNAs in different groups vs. non-users included 2223 in e-cig users, 3244 in cigarette smokers, 2428 in waterpipe smokers, and 2887 in dual smokers. Many of the identified target genes were unique to a specific group; however, there were also common target genes across different groups [110].

We performed RNA-seq to profile the whole transcriptome of oral epithelial cells in healthy adult “exclusive” vapers (*N* = 42), “exclusive” cigarette smokers (*N* = 24), and non-users (*N* = 27) [19]. The choice of oral epithelial cells for transcriptome analysis was based on the following: (1) the oral epithelium is the first site of “direct” exposure to the toxic and carcinogenic compounds present in e-cig vapor and cigarette smoke [112,113,114,115,116]; (2) oral epithelial cells are a major target for tumorigenesis and other anomalies associated with tobacco use [117,118]; (3) more than 90% of all human cancers are of epithelial origin [119,120]; (4) oral epithelial cells and lung epithelial cells show remarkable similarities in response to respiratory toxicants and carcinogens, as evidenced by the comparable patterns of genotoxic [121,122,123,124,125,126,127,128,129,130], epigenetic [131,132,133,134], and transcriptomic effects [135,136,137,138,139] in smokers’ oral cells and lung cells, respectively; and (5) oral epithelial cells are readily available for sampling using non-invasive methods [112,137,140].

**Figure 3:**
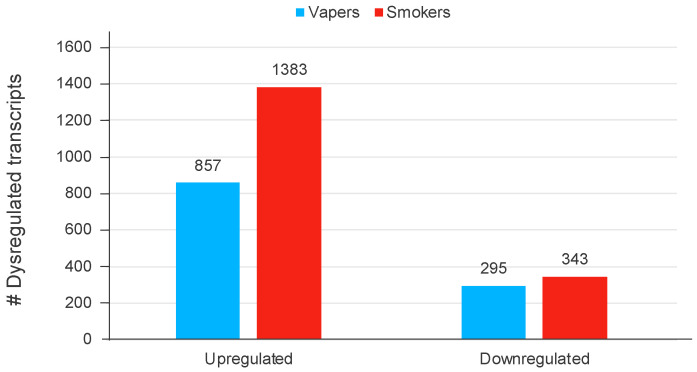
Differentially expressed transcripts in the oral epithelial cells of vapers and smokers compared to non-users. Numbers of upregulated and downregulated gene transcripts in vapers and smokers compared to non-users are shown. Data are derived from ref. [19].

We detected large sets of aberrantly expressed gene transcripts in both vapers and smokers compared to non-users; however, smokers showed nearly 50% more dysregulated transcripts than vapers (1726 vs. 1152) (Figure 3). Of note, 47% of the aberrantly expressed transcripts in vapers were non-coding (vs. 21% in smokers).

**Figure 4:**
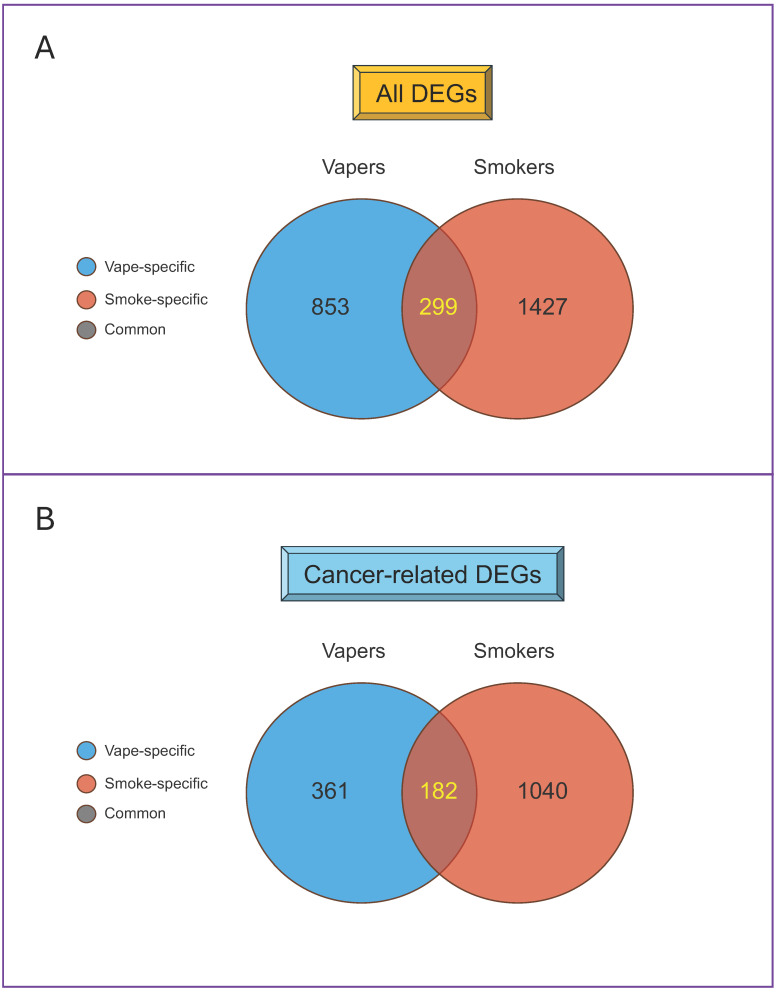
Differentially expressed genes in the oral epithelial cells of vapers and smokers compared to non-users. Venn diagrams of all dysregulated genes (**A**) and cancer-related dysregulated genes (**B**) in vapers and smokers compared to non-users. DEGs = differentially expressed genes. Data are derived from ref. [19].

As shown in Figure 4A, the dysregulated transcripts in vapers and smokers can be classified as follows: (1) vape-specific (853 transcripts); (2) smoke-specific (1427 transcripts); and (3) common to vape and smoke (299 transcripts). Gene ontology analysis showed that cancer was the top disease associated with the differentially expressed genes (DEGs) in both vapers (62%) and smokers (79%). The cancer-related DEGs included 361 genes specific to vapers, 1040 genes specific to smokers, and 182 genes common to vapers and smokers (total: 1583) (Figure 4B). The DEGs in both vapers and smokers were also associated with other diseases and conditions, specifically inflammation. Of note, some of the DEGs in vapers and smokers are known to be frequently targeted in the early stages of diseases, such as oral epithelial dysplasia, that can progress to malignancy [52].

Molecular pathway and functional network analysis of the DEGs revealed that the “Wnt/Ca^+^ pathway” was the most affected pathway in vapers, whereas the “integrin signaling pathway” was the most disrupted pathway in smokers [19]. The Wnt/Ca^2+^ signaling pathway, which is activated by the tumor suppressor *WNT5A* in the presence of a “frizzled” class receptor, is known to be downregulated in several types of human cancer [141]. Of significance, the *WNT5A* gene and the frizzled receptor *FDZ7* gene were both downregulated in vapers, likely inhibiting the downstream effectors of the cascade. The integrin signaling pathway is shown to control cell proliferation, survival, and migration. When dysregulated, the integrin signaling pathway can promote tumor invasion and metastasis [142]. The top dysregulated pathway common to vapers and smokers was the “Rho family GTPases signaling pathway”, although the number of affected targets was three times greater in smokers than vapers (27 vs. 9) [19]. The GTPase family of small GTP-binding proteins includes a group of signaling molecules, which are activated by growth factors, hormones, integrins, cytokines, and adhesion molecules [143]. They regulate the reorganization of the actin cytoskeleton, transcriptional regulation, vesicle trafficking, morphogenesis, neutrophil activation, phagocytosis, mitogenesis, apoptosis, and tumorigenesis. Rho GTPases are also implicated in the DNA-damage response consequent to assault by genotoxic compounds [143].

Currently, work in our laboratory is underway to perform miRNA-seq on the collected specimens from vapers, smokers, and non-users. We will identify dysregulated miRNAs in each group and conduct an integrative analysis of the miRNA and mRNA data in vapers and smokers (ongoing work).

## 5. Conclusions and Perspectives

Accumulating evidence shows that miRNAs play a key role in the regulation of gene expression in health and disease states [31,32,33,34,35]. MiRNAs have exhibited great promise as biomarkers of exposure and effects for disease-causing agents [40,41,42,43,44,45,46]. A limited but growing number of studies have demonstrated the utility of miRNAs for investigating the biological effects of vaping [19,110,111]. The existing data show that the differential expression of miRNAs and the dysregulation of disease-related genes are detectable in the cells and tissues of vapers [19,110] as well as in cells treated in vitro with e-cig liquid or vapor [111]. The dysregulated genes and associated molecular pathways and gene networks in vapers have been shown to be partly similar to and partly different from those found in smokers [19,110]. The differentially expressed miRNAs and associated target genes common to vapers and smokers can be ascribed to the shared exposure of both groups to chemicals present in e-cig vapers and tobacco smoke [19,79,144]. Conversely, the unique dysregulated miRNAs and associated target genes in vapers or smokers can be attributed to the specific exposure of each group to chemicals present only in e-cig vapor or tobacco smoke [19,67,144]. Dual users of e-cigs and combustible tobacco products have shown dysregulation of some of the same differentially expressed miRNAs and associated target genes as those found in exclusive vapers and exclusive smokers [110]. Nevertheless, dual users have also shown unique dysregulated miRNAs and associated target genes that likely stem from the interactive effects of the combined use of e-cigs and combustible tobacco products [55,110].

It is important to note that the (relatively) small size of the studied populations has precluded the examination of the contribution of product characteristics to the observed effects in vapers [19,110]. Product characteristics of paramount importance include e-cig device type, device features, and e-liquid content (i.e., flavor type, nicotine concentration, humectants). Future studies with large sample sizes and high statistical power should enable the determination of the role played by product characteristics in miRNA-mediated dysregulation of disease-related genes in vapers. Follow-up investigations should compare and contrast the expression profiles of miRNA in diverse and preferably non-invasively obtainable specimens from e-cig users while factoring in the dynamic changes and uniformities in epigenetic marks across different cell types [17,18,131,145,146]. Furthermore, the effect of local vs. systemic exposure to chemical constituents of e-cig vapor should be taken into account when analyzing different cells and tissues from e-cig users, e.g., oral or nasal epithelia vs. peripheral blood.

Since many transcriptomic changes occur in the early stages of disease—often preceding clinical manifestation of the disease [147,148,149]—one should expect to find many dysregulated disease-related coding and non-coding genes, e.g., miRNAs in apparently healthy vapers, smokers, and dual users, as shown by us [19,27,144] and others [110]. The affected miRNAs and associated target genes in healthy vapers and/or smokers are likely to be dysregulated to a lower extent than those in patients diagnosed with diseases. The cumulative exposure of chronic vapers and smokers to large quantities of toxicants and carcinogens present in e-cig vapor and tobacco smoke should result in transcriptomic changes, such as aberrant miRNA and mRNA expression, similar to those found in patient populations, although patients are likely to have more pronounced expression changes. The dysregulated miRNAs and associated target genes found in vapers and/or smokers have been linked to diseases like cancer, respiratory diseases, cardiovascular diseases, and/or immune diseases [19,110,111]. The same diseases are caused by or associated with tobacco product use [1,2,6,7,13,14,27,28,30,144,150,151,152].

Because a single miRNA can have multiple gene targets and an individual gene can be targeted by many miRNAs [31,32,35], most dysregulated miRNAs in vapers and/or smokers are expected to be associated with multiple diseases. To remove noise from data, statistical and bioinformatic approaches should be employed to prioritize miRNA–disease pairs with the highest association specificity and sensitivity in vapers and/or smokers. Finally, although association studies of molecular changes and disease are increasingly used for biomarker discovery in humans [147,153], follow-up functional studies involving miRNA mimics [154], antisense oligonucleotides [155], and high throughput 3′ untranslated region (3′-UTR) reporter assays in cell lines [156] should be conducted to verify whether the detectable differentially expressed miRNAs in vapers and/or smokers are causally involved in disease pathogenesis.

## Figures and Tables

**Table 1:** List of top 20 differentially expressed miRNAs in primary human bronchial epithelial cells treated with non-vaporized or vaporized e-cig liquid, with or without nicotine (*N* = 12), compared to untreated controls (*N* = 3) from the Solleti et al. study (ref. [111]).

MiRNA	Observed SAM Score (d) *	MiR-Target Network ^†^
*hsa-miR-421* ^‡^	−8.84	831 predicted targets ^§^
*hsa-miR-23a-3p*	−8.75	*TSC1*, *EN2*, *PPARGC1A*, *ADAM28*, *SMAD5*, *SOD2*, *LMAN2L*, *PTEN*, *SDHD*, *CHD4*, *PTPN11*, *TRPM7*, *ABCD1*, *ALDH5A1*, *SKI*, *PSAP*, *STAT3*, *STS*, *CXCL12*, *LDHA*, *CLDN16*, *NAV2*, *FAS*, *TNFAIP3*, *CCL8*, *IL6R*, *IRF1*, *GJA1*, *TSNAX*, *NUFIP2*, *RGS5*, *AMBRA1*, *KLF12*, *TGFB2*, *FKBP5*, *IKZF3*, *SLC1A5*, *MC2R*, *RFC2*, *CXCL8*, *ADK*, *LPAR1*, *MOG*, *C9orf3*, *NLGN4X*, *TBL2*, *SMAD3*, *MEF2C*, *PEX26*
*hsa-miR-186-5p*	−8.47	*SOD2*, *MATR3*, *RAF1*, *CNBP*, *CDKN1B*, *TOP2A*, *EEF2*, *DPP9*, *TNFSF15*, *KIF6*, *WNT5A*, *CPT1A*, *FGFR2*, *MYH9*, *ATM*, *ACVR1*, *HMGB1*, *OLR1*, *PTGIS*, *MAPK14*, *VEGFA*, *WDR11*
*hsa-miR-140-5p*	−7.4	*GDNF*, *SKI*, *ARID5B*, *SUZ12*, *FGF9*, *ADA*, *FGF2*, *CDK6*, *SHANK3*, *PRDM1*, *TPI1*, *NFYA*, *FPGS*, *SLC7A5*, *ITGA6*, *VEGFA*
*hsa-let-7f-5p*	−6.61	*CCNG1*, *HDAC2*, *EPHA4*, *SMARCC1*, *ATXN2*, *EDN1*, *IKZF3*, *CYP19A1*, *BAZ1B*, *IL6*, *GLUL*, *CRX*, *IL13*
*hsa-miR-28-5p*	−6.56	*TPM4*, *TUFM*, *TP53*
*hsa-miR-130a-3p*	−6.55	*PPARGC1A*, *ADAM28*, *SOD2*, *PTPN14*, *SMAD4*, *ZBTB7A*, *CCND2*, *PTEN*, *MET*, *ABCG8*, *LDLR*, *COX10*, *TGFBR1*, *TGFBR2*, *IFNLR1*, *RNF125*, *ARSA*, *LIPA*, *MECP2*, *FKTN*, *IL23R*, *CTSA*, *SLC31A1*, *MAPK1*, *P2RY11*, *PPARA*, *PPARG*, *IER3IP1*, *QKI*, *APP*, *XIAP*, *MC2R*, *ESR1*, *SATB2*, *TPP1*, *CLCN3*, *PEX13*, *RUNX3*
*hsa-miR-98-5p*	−6.42	*ABCC4*, *PPARGC1A*, *FZD3*, *CD99*, *CCND2*, *CYP19A1*, *CHST3*, *PDLIM5*, *EDN1*, *IGF1*, *CREB1*, *IL6R*, *CHD7*, *SLC31A1*, *CDKN1A*, *THBS1*, *CAP1*, *LIFR*, *PLPP3*, *CDKN2B*, *TRAF1*, *VCL*, *REL*, *SIGMAR1*, *MPL*, *LGR4*, *KREMEN1*, *ICAM1*, *IGF2BP2*, *ZMPSTE24*
*hsa-miR-151a-3p*	−6.26	*TP53*, *ZBTB24*, *SLC6A1*, *MLH3*, *MPL*, *NOP10*
*hsa-miR-182-5p*	−6.11	*NDRG1*, *BDNF*, *CDKN1B*, *FGF9*, *FBXW7*, *PTEN*, *MITF*, *BARD1*, *PSD3*, *IGF1R*, *GSK3B*, *CREB1*
*hsa-miR-1307-3p* ^‡^	8.33	*VPS37C*, *CIC*, *TAGLN3*, *CCDC28B*, *AGAP1*, *ISM1*, *PRKCZ*
*hsa-miR-146b-3p*	8.34	*SQSTM1*, *KCTD15*, *CDKN1A*, *ERBB4*, *NUFIP2*, *NPAS4*, *REL*, *EGFR*, *SLC5A5*, *MYLK*, *KIT*, *IL6*, *TLR4*, *FAM107A*
*hsa-miR-25-5p*	8.57	*CDKN1C*, *NFIX*, *TP53*, *MDM4*, *ERBB2*, *NF2*, *SH2B3*, *14-Sep*, *TNFSF10*, *FGF2*, *XPC*, *CDH1*, *MDM2*, *NOTCH2*, *RECK*, *DHFR*, *NRAS*, *TNFRSF10A*
*hsa-miR-148b-3p*	9.42	*PIK3CA*, *CYP1B1*, *CCL11*, *AHI1*, *PIK3CG*, *HMOX1*, *SRSF10*, *HLA-A*, *MCL1*, *MECP2*, *HLA-G*, *C3orf58*, *RUNX3*
*hsa-miR-320a/b/c/d* ^‡^	10.13	(a) *TSC1*, *TFAP2A*, *GAPDH*, *RPS6KA3*, *CDKN2A*, *MOCS1*, *PTEN*, *CCND2*, *PRDX3*, *MET*, *DIAPH1*, *PLS3*, *BRD4*, *CYBA*, *RNF125*, *KMT2D*, *TPI1*, *IGF2*, *CLN6*, *MMP9*, *FAS*, *KDM5C*, *YWHAE*, *GLUL*, *PPT1*, *HSD17B10*, *MDK*, *HNF1A*, *MLX*, *XBP1*, *CDK6*, *PKM*, *ATP7A*, *AR*, *TUBA1B*, *PACS1*, *NCOR1*, *RERE*, *SLC6A8*, *EZH2*, *VDAC1*, *VEGFA*, *RUNX2* (b) 1045 predicted targets ^§^ (c) 1045 predicted targets ^§^ (d) *MDK*
*hsa-miR-342-5p*	12.51	*ACOX1*, *JUN*, *PFN1*, *FOSL2*, *NAA10*, *ZBTB7A*, *CCND2*, *COX10*, *DIAPH1*, *FAM107A*, *SKI*, *KCTD15*, *PLA2G4A*, *IDS*, *MMACHC*, *GGCX*, *MDM4*, *NDRG1*, *TFRC*, *IKBKG*, *CDK6*, *FXN*, *CLN8*, *ENG*, *ANTXR2*, *IKZF3*, *PPM1L*, *MAF*, *ACTG1*, *ABL1*, *PPM1D*, *IGF2R*, *ELN*, *UCP3*, *PTGIS*, *VAPB*, *IGF1R*
*hsa-miR-887-3p* ^‡^	12.9	*CASK*, *CCPG1*, *PLD2*, *TCTN3*, *ATP2B2*, *CNPY2*, *C3orf80*, *FARP1*, *NPAT*, *CRLF3*, *MORC3*, *STARD13*, *POM121*, *TMEM139*, *TXNDC5*, *ST6GAL2*, *TMEM38B*, *PLEKHG2*, *MAP3K1*, *NCOR2*, *FADS6*, *CALM1*, *NT5C3A*, *FBXO34*, *RINL*, *PRH2*, *GSK3A*, *GAP43*, *HNRNPA3*, *GATD1*, *RRP36*, *GCNT2*
*hsa-miR-7706* ^‡^	13.63	*TFAP2A*, *BLCAP*, *IQGAP1*, *CSNK2A1*, *ZNF468*, *ALAS2*, *ZBTB20*, *ANO8*, *ADO*, *SPNS2*, *FOSB*, *CTDSPL2*, *PWWP3B*, *RAB29*, *CELF4*, *TMEM65*, *DOCK4*, *RER1*, *CCDC103*, *GAS6*, *COMMD8*, *COL9A1*, *PCDHGA11*, *PCDHGA2*, *PCDHGC5*, *PCDHGA12*, *PCDHGB5*, *PCDHGA1*, *PCDHGA8*, *PCDHGA7*, *PCDHGB3*, *PCDHGB2*, *PCDHGA9*, *PCDHGB7*, *PCDHGA10*, *PCDHGC4*, *SELENOW*, *PCDHGC3*, *PCDHGB1*, *PCDHGA3*, *PCDHGB4*, *PCDHGA5*, *PCDHGA4*, *PCDHGB6*, *PCDHGA6*, *MZF1*, *KPNA1*, *DUSP18*, *PHTF1*, *KDM5A*, *PMF1-BGLAP*, *ZKSCAN3*, *ACTN2*, *INPP5A*, *GEMIN7*, *PPP1R9A*, *GNG12*, *DCP2*, *PMEPA1*, *CALHM3*, *FNBP1*, *GLG1*, *ATF6*, *CITED2*, *WNK1*, *DPYSL5*, *CCDC93*, *RETREG3*, *NFIX*, *SNX9*, *ATRX*, *MBOAT2*, *TGFBR3L*, *FCHSD1*, *ZNF385A*, *ZEB1*, *PAX6*
*hsa-miR-130b-5p*	13.96	*PIK3CB*, *ARHGAP1*, *PTMA*, *DICER1*, *PPARGC1A*, *GRIN2A*, *PARP1*, *JAG2*, *SLC38A2*, *ADARB1*, *ZBTB7A*, *CCND2*, *PTEN*, *JARID2*, *MLXIPL*, *SLC30A3*, *RIMS3*, *LDLR*, *TCF7L2*, *TGFBR2*, *RB1*, *NR3C1*, *MMP2*, *LIPA*, *SH3PXD2A*, *WNK3*, *EDN1*, *HRH1*, *IGF1*, *RASSF1*, *RAPGEF6*, *SYNGR1*, *PLXNA3*, *SLC31A1*, *FMR1*, *MBNL1*, *TFRC*, *MAPK1*, *TES*, *PPARA*, *PLP1*, *PPARG*, *TCF4*, *SLC26A7*, *QKI*, *XIAP*, *LRP8*, *PSD3*, *EIF2S1*, *RFC2*, *CBLN2*, *CD86*, *SHOX*, *ACVR1*, *NRN1*, *ESR1*, *KMT2A*, *PLEKHA6*, *EPHA4*, *OXSR1*, *NOTCH2*, *CCNA2*, *EMX2*, *KREMEN1*, *CLCN3*, *CHEK2*, *ACSL4*, *RUNX3*
*hsa-miR-941* ^‡^	14.01	*ZNF324*, *CROT*, *ATP8B2*, *FBXO42*, *PRDM16*, *PGP*, *ANKRD34B*, *CKS1B*, *NDUFAF7*, *SERTAD3*, *HINT3*, *MTMR9*, *CACNG8*, *RAI1*, *SPSB1*, *TMEM203*, *SAV1*, *CLCN3*, *KCMF1*, *POLH*, *SAR1A*, *RGS10*, *ORAI2*, *FOXN4*, *VPS53*, *ASAH2B*, *SLC35E4*, *SYNRG*

Data are derived from ref. [111]. SAM-Seq analysis (at median false discover rate (FDR) = 0) identified 578 significantly differentially expressed miRNAs in cells treated with any e-cig liquid compared to untreated controls. For brevity, the top 20 differentially expressed miRNAs, including 10 upregulated and 10 downregulated miRNAs, are shown. A full list of all 578 differentially expressed miRNAs is available in Supplemental Table S1 in ref. [111]. * A negative SAM score indicates an increase in expression when treated with e-cig liquid. A positive SAM score indicates a decrease in expression when treated with e-cig liquid. ^†^ For each miRNA, a network of miRNA–target interactions (disease–context), based on the experimentally supported miRNA–target data from miRTarBase (https://mirtarbase.cuhk.edu.cn/ (accessed on 3 March 2024)), is provided using the Human microRNA Disease Database version 4.0 (HMDD v.4.0) (http://www.cuilab.cn/hmdd (accessed on 3 March 2024)). ^‡^ For those miRNAs that have not been entered into HMDD v.4.0, predicted targets are indicated according to the miRDB database (https://mirdb.org/ (accessed on 3 March 2024)). ^§^ Due to space limits, the number of the predicted targets is indicated. Full descriptions of the predicted targets, including target detail, target rank, target score, gene symbol, and gene description, are available at: https://mirdb.org/ (accessed on 3 March 2024).

**Table 2:** List of differentially expressed miRNAs in plasma exosomes of e-cig users (*N* = 7) compared to non-users (*N* = 8) from the Singh et al. study (ref. [110]).

MiRNA	Log2 Fold Change	*t*-Test *p* Value	FDR Adjusted *p* Value	MiR-Target Network *
*hsa-miR-365a/b-3p*	24.32 ↑	2.49 × 10^−34^	1.18 × 10^−31^	(a) *CDKN1A*, *DOK2*, *AKT1*, *CCND1*, *KRAS*, *MTA2*, *IL7R* (b) *DOK2*, *AKT1*, *IL7R*, *CDKN1A*
*hsa-miR-362-5p*	−44.56 ↓	9.82 × 10^−23^	2.32 × 10^−20^	*CASP8*, *FGF9*
*hsa-miR-29b-3p*	−24.34 ↓	2.75 × 10^−17^	4.33 × 10^−15^	*FBN1*, *REST*, *LOX*, *MDM2*, *TET2*, *CNBP*, *SMARCC1*, *LAMA2*, *COL5A1*, *BCL2*, *TNFAIP3*, *DNMT3A*, *TGFB2*, *VHL*, *IFNG*, *ESR1*, *BACE1*, *CCNA2*, *NOTCH2*, *HDAC4*, *AQP4*
*hsa-let-7f-5p*	1.41 ↑	9.74 × 10^−8^	1.15 × 10^−5^	*CCNG1*, *HDAC2*, *EPHA4*, *SMARCC1*, *ATXN2*, *EDN1*, *IKZF3*, *CYP19A1*, *BAZ1B*, *IL6*, *GLUL*, *CRX*, *IL13*
*hsa-miR-1299* ^†^	20.10 ↑	1.50 × 10^−7^	1.42 × 10^−5^	1119 predicted targets ^§^
*hsa-miR-21-5p*	1.30 ↑	7.13 × 10^−7^	5.29 × 10^−5^	*LATS1*, *DICER1*, *MIB1*, *PTPN14*, *REST*, *SLC17A5*, *RPS6KA3*, *GDF5*, *NR2C2*, *IGF1R*, *TGFBR2*, *CYCS*, *STAT3*, *RB1*, *COL4A1*, *PTPN3*, *OXTR*, *SOX11*, *CCL1*, *CADM1*, *LAMP2*, *DMD*, *CLCN5*, *BAZ1B*, *SLC9A6*, *GGCX*, *BCL2*, *TOP2A*, *KAT6A*, *KLF9*, *MDM4*, *PTGFR*, *SLC31A1*, *ZBTB20*, *FMR1*, *FUT2*, *SEMA5A*, *CCNG1*, *HS3ST3B1*, *PURA*, *KIF6*, *CCND1*, *PPARA*, *NBEA*, *CDK6*, *LIFR*, *TCF21*, *WNT5A*, *FKBP5*, *SOX5*, *RECK*, *PLAT*, *TRIM44*, *EIF2S1*, *TLR4*, *PPM1L*, *GTF2I*, *CEP152*, *AGAP1*, *NTF3*, *FOXO3*, *HPGD*, *CPM*, *HMGB1*, *EGFR*, *PIK3R1*, *GNE*, *RP2*, *NIPBL*, *TIMP3*, *SOX2*, *BMI1*, *MUC1*, *PREPL*
*hsa-let-7i-5p*	1.37 ↑	7.84 × 10^−7^	5.29 × 10^−5^	*MDM4*, *MYBPC3*, *SOD2*, *EPHA4*, *CCND1*, *EDN1*, *IKZF3*, *ACTA1*, *IGF1*, *IGF1R*, *MAP2K7*, *CRX*, *IL13*
*hsa-let-7a-5p*	1.53 ↑	1.52 × 10^−6^	8.96 × 10^−5^	*MYC*, *ARG2*, *MDM2*, *SIK1*, *F2R*, *CRX*, *IFNLR1*, *CASP3*, *EDN1*, *IGF2*, *BCL2*, *AP1S1*, *MDM4*, *NPC1*, *THBS1*, *CDKN1A*, *DUSP6*, *CCNG1*, *TES*, *KRAS*, *CDK6*, *FXN*, *IKZF3*, *BTG1*, *EPHA4*, *VCL*, *MPL*, *ACTA1*
*hsa-miR-30a-5p*	1.50 ↑	1.56 × 10^−5^	0.000735	*DGKH*, *DROSHA*, *TP53*, *CTNNB1*, *PPARD*, *SOD2*, *PRKAR1A*, *FBXO45*, *SLC38A2*, *ELOVL5*, *MAPK8*, *NPTN*, *MET*, *LDLR*, *OPHN1*, *HSPA5*, *ESR2*, *SLC1A2*, *PPARGC1B*, *FOXG1*, *BCL11A*, *CREM*, *CASP3*, *MPDU1*, *SH3PXD2A*, *GNAL*, *MECP2*, *MTR*, *PEX11B*, *SLC7A5*, *YWHAE*, *EEF2*, *ITGB3*, *CNP*, *THBS1*, *MAPK1*, *NUFIP2*, *NCAM1*, *PDCD10*, *KRAS*, *ATRX*, *CDK6*, *LIFR*, *WNT5A*, *ENTPD4*, *HDAC1*, *SCML2*, *PNPO*, *KCNN3*, *TGM2*, *KPNA1*, *KMT2A*, *SP4*, *EGFR*, *NDE1*, *PBRM1*, *KREMEN1*, *PPP3R1*, *IGF1R*, *RUNX2*, *PREPL*
*hsa-miR-193b-3p*	8.77 ↑	1.41 × 10^−5^	0.000735	*CS*, *TSC1*, *GUCA1B*, *PARP1*, *SYNE2*, *FANCD2*, *CHD4*, *DNMT1*, *OPHN1*, *CYCS*, *PPARGC1B*, *NAGA*, *DLX1*, *LASP1*, *AKT1*, *FANCA*, *GLO1*, *GJB2*, *MTR*, *GPR155*, *HIST1H3B*, *BAZ1B*, *STMN1*, *KAT6A*, *FBXL19*, *WDPCP*, *SRSF10*, *KRAS*, *CDK6*, *NF1*, *ARID1A*, *ESR1*, *SMAD3*, *MYLK*, *RAC2*
*hsa-miR-100-5p*	1.25 ↑	8.37 × 10^−5^	0.003478	*AKT1*, *FKBP5*, *RB1*, *IGF1R*
*hsa-miR-423-3p*	1.48 ↑	8.84 × 10^−5^	0.003478	*PTMA*, *PML*, *FASN*, *CDKN2A*, *PPP2R1B*, *MAPK3*, *MTCH2*, *CSTB*, *KCTD15*, *PPARGC1B*, *RUNX1*, *BAZ1B*, *GLUL*, *FBXL19*, *CREB1*, *IRAK1*, *MDM4*, *NDRG1*, *CDKN1A*, *CALR*, *MTHFR*, *PFKFB3*, *GJB1*, *HLA-C*, *OLR1*, *VEGFA*
*hsa-miR-30c-5p*	1.50 ↑	0.000276283	0.010031	*TP53*, *SERPINE1*, *PPARGC1B*, *CTGF*, *NOTCH1*, *SUZ12*, *LIFR*, *MCL1*, *SLC7A5*, *LDLR*
*hsa-miR-451a* ^†^	−1.80 ↓	0.000781282	0.02634	*OSR1*, *CUX2*, *PSMB8*, *CXCL16*, *TARP*, *ST8SIA4*, *CDKN2D*, *MIF*, *FBLN5*, *CERK*, *SAMD4B*, *CAB39*, *VAPA*, *LETM2*, *MEX3C*, *USP46*, *CMTM6*, *TBC1D9B*, *PMM2*, *KIAA1217*, *MAU2*, *RNF217*, *MEGF6*, *S1PR2*, *EVL*, *FBXO33*, *ATF2*, *CDKN2B*, *UCK1*, *CAV1*, *C16orf72*, *DCAF5*, *RAB5A*, *CACHD1*, *LUZP2*, *EIF2AK3*, *AKTIP*, *FAM171A1*, *TTN*, *NEDD9*
*hsa-miR-143-3p*	1.04 ↑	0.00091283	0.028724	*PAPPA*, *ADCY2*, *STAR*, *MDM2*, *NR2C2*, *MMP14*, *CNBP*, *TRAF3IP2*, *MMP2*, *AKT1*, *IDS*, *COL5A1*, *MMP9*, *THRA*, *GLUL*, *TNF*, *IL2RA*, *IRF1*, *MAPK1*, *DNMT3A*, *KRAS*, *IKZF3*, *XIAP*, *ITGB1*, *PTPN2*, *TEP1*, *PTGS2*, *PIK3R1*, *SMAD3*, *SMYD4*, *FHIT*, *LIMK1*, *IGF1R*
*hsa-miR-224-5p*	2.01 ↑	0.001192386	0.035175	*PPP2CA*, *CCND1*, *SMAD4*, *KRAS*, *MKKS*, *MDM2*, *QKI*, *BCL2*
*hsa-miR-30e-5p*	−1.30 ↓	0.001717024	0.047673	*DGKH*, *TP53*, *PRKAR1A*, *MKRN3*, *RELA*, *LDLR*, *SLC1A2*, *PPARGC1B*, *CASP3*, *SH3PXD2A*, *GLO1*, *MTR*, *YWHAE*, *MDM4*, *AFF4*, *MBNL1*, *NUFIP2*, *KRAS*, *BTBD9*, *KCNN3*, *ATP2A2*, *DGCR8*, *PBRM1*, *KREMEN1*, *BMI1*

Data are derived from ref. [110]. Arrows indicate upregulated (↑) miRNAs and downregulated (↓) miRNAs. FDR = False discovery rate. * For each miRNA, a network of miRNA–target interactions (disease–context), based on the experimentally supported miRNA–target data from miRTarBase (https://mirtarbase.cuhk.edu.cn/ (accessed on 3 March 2024)), is provided using the Human microRNA Disease Database version 4.0 (HMDD v.4.0) (http://www.cuilab.cn/hmdd (accessed on 3 March 2024)). Upregulated target genes of miRNAs are in blue color font and downregulated target genes of miRNAs are in black color font. ^†^ For those miRNAs that have not been entered into HMDD v.4.0, predicted targets are indicated according to the miRDB database (https://mirdb.org/ (accessed on 3 March 2024)). ^§^ Due to space limits, the number of the predicted targets is indicated. Full descriptions of the predicted targets, including target detail, target rank, target score, gene symbol, and gene description, are available at: https://mirdb.org/ (accessed on 3 March 2024).

## Data Availability

All data are contained within the article.

## Supplementary Materials

The following supporting information can be downloaded at: https://www.mdpi.com/article/10.3390/cells13161330/s1, Table S1: List of differentially expressed miRNAs in plasma exosomes of cigarette smokers (*N* = 7) as compared to non-users (*N* = 8) from the Singh et al. study (ref. [110]); Table S2: List of differentially expressed miRNAs in plasma exosomes of waterpipe smokers (*N* = 7) as compared to non-users (*N* = 8) from the Singh et al. study (ref. [110]); and Table S3: List of differentially expressed miRNAs in plasma exosomes of dual smokers (both cigarettes and waterpipe, *N* = 7) as compared to non-users (*N* = 8) from the Singh et al. study (ref. [110]).
