# Supplementary material for: The Untapped Biomarker Potential of MicroRNAs for Health Risk–Benefit Analysis of Vaping vs. Smoking
Source: Cells. 2024 Aug 10;13(16):1330. doi: 10.3390/cells13161330 (PMC11352591; doi:10.3390/cells13161330)
Supplement: Supplementary file 1 [file cells-13-01330-s001.zip › Table S2.pdf]

Supplementary Table S2. List of differentially expressed miRNAs in plasma exosomes of waterpipe smokers (N = 7) as compared to non-users (N = 8) from the Singh *et al*. study (ref. [110]).

miRNA	Log2 fold change	t-test *p*-value	FDR adjusted *p*-value	miR-Target Network *
hsa-miR-2355-5p †	39.82 ↑	2.05E-26	9.67E-24	767 predicted targets §
hsa-miR-362-5p	-45.36 ↓	1.67E-23	3.95E-21	*CASP8, FGF9*
hsa-miR-29b-3p	-24.6 ↓	1.19E-17	1.87E-15	*FBN1, REST, LOX, MDM2, TET2, CNBP, SMARCC1, LAMA2, COL5A1, BCL2, TNFAIP3, DNMT3A, TGFB2, VHL, IFNG, ESR1, BACE1, CCNA2, NOTCH2, HDAC4, AQP4*
hsa-miR-582-5p	23.15 ↑	7.50E-11	8.85E-09	*MCL1*
hsa-miR-149-5p	29.22 ↑	1.31E-10	1.24E-08	*TP53, FOSL2, FPGS, LDLR, MAP2K7, CNBP, CALD1, FASLG, HECTD4, AKT1, CDKN1B, HLA-A, TRAF6, AHR, BCL2L1, SLC7A5, CD40LG, BIRC5, IGF1, ITGB3, CDKN1A, ADIPOQ, MLX, TES, YARS2, CALR, MTHFR, IKZF3, IL6, TRIM44, GPRC5A, MYH9, SNRNP200, FGFR1, KIF1A, AIP, OGG1, MYD88*
hsa-miR-1299 †	19.62 ↑	2.96E-07	2.33E-05	1,119 predicted targets §
hsa-miR-1-3p	7.57 ↑	7.31E-07	4.93E-05	*SYNE1, UST, HMOX1, HDAC2, CXCL12, SMARCC1, RASSF1, GJA1, CCND1, BAX, CDK6, RARB, RASSF5, CALR, SOX5, PRKCE, IL6, TLR4, PIK3CA, DDX5*
hsa-let-7i-5p	1.09 ↑	8.84E-05	0.005213	*MDM4, MYBPC3, SOD2, EPHA4, CCND1, EDN1, IKZF3, ACTA1, IGF1, IGF1R, MAP2K7, CRX, IL13*
hsa-miR-320b †	-2.30 ↓	0.000226	0.01184	1,045 predicted targets §
hsa-miR-21-5p	0.93 ↑	0.000384	0.015101	*LATS1, DICER1, MIB1, PTPN14, REST, SLC17A5, RPS6KA3, GDF5, NR2C2, IGF1R, TGFBR2, CYCS, STAT3, RB1, COL4A1, PTPN3, OXTR, SOX11, CCL1, CADM1, LAMP2, DMD, CLCN5, BAZ1B, SLC9A6, GGCX, BCL2, TOP2A, KAT6A, KLF9, MDM4, PTGFR, SLC31A1, ZBTB20, FMR1, FUT2, SEMA5A, CCNG1, HS3ST3B1, PURA, KIF6, CCND1, PPARA, NBEA, CDK6, LIFR, TCF21, WNT5A, FKBP5, SOX5, RECK, PLAT, TRIM44, EIF2S1, TLR4, PPM1L, GTF2I, CEP152, AGAP1, NTF3, FOXO3, HPGD, CPM, HMGB1, EGFR, PIK3R1, GNE, RP2, NIPBL, TIMP3, SOX2, BMI1, MUC1, PREPL*
hsa-let-7f-5p	0.94 ↑	0.000378	0.015101	*CCNG1, HDAC2, EPHA4, SMARCC1, ATXN2, EDN1, IKZF3, CYP19A1, BAZ1B, IL6, GLUL, CRX, IL13*
hsa-miR-143-3p	1.12 ↑	0.000332	0.015101	*PAPPA, ADCY2, STAR, MDM2, NR2C2, MMP14, CNBP, TRAF3IP2, MMP2, AKT1, IDS, COL5A1, MMP9, THRA, GLUL, TNF, IL2RA, IRF1, MAPK1, DNMT3A, KRAS, IKZF3, XIAP, ITGB1,*

				PTPN2, TEP1, PTGS2, PIK3R1, SMAD3, SMYD4, FHIT, LIMK1, IGF1R
hsa-miR-320d	-4.58 ↓	0.000509	0.018471	*MDK*
hsa-let-7a-5p	1.02 ↑	0.001386	0.045733	*MYC, ARG2, MDM2, SIK1, F2R, CRX, IFNLR1, CASP3, EDN1, IGF2, BCL2, AP1S1, MDM4, NPC1, THBS1, CDKN1A, DUSP6, CCNG1, TES, KRAS, CDK6, FXN, IKZF3, BTG1, EPHA4, VCL, MPL, ACTA1*
hsa-miR-30a-5p	1.10 ↑	0.00155	0.045733	*DGKH, DROSHA, TP53, CTNNB1, PPARD, SOD2, PRKAR1A, FBXO45, SLC38A2, ELOVL5, MAPK8, NPTN, MET, LDLR, OPHN1, HSPA5, ESR2, SLC1A2, PPARGC1B, FOXG1, BCL11A, CREM, CASP3, MPDU1, SH3PXD2A, GNAL, MECP2, MTR, PEX11B, SLC7A5, YWHAE, EEF2, ITGB3, CNP, THBS1, MAPK1, NUFIP2, NCAM1, PDCD10, KRAS, ATRX, CDK6, LIFR, WNT5A, ENTPD4, HDAC1, SCML2, PNPO, KCNN3, TGM2, KPNA1, KMT2A, SP4, EGFR, NDE1, PBRM1, KREMEN1, PPP3R1, IGF1R, RUNX2, PREPL*
hsa-let-7g-5p	0.93 ↑	0.00155	0.045733	*NFIX, SOD2, MBD2, MAP2K7, PDLIM5, CRX, CASP3, DISC1, NDUFS1, HMGA1, BCL2L1, IL13, MDM4, IL6R, THBS1, FYN, CCND1, KRAS, IKZF3, MAP3K1, ARID1A, EPHA4, HMGB1,OLR1, KREMEN1, RHD*

Data are derived from ref. [110]. Arrows indicate upregulated (↑) miRNAs and downregulated (↓) miRNAs. FDR = False discovery rate

* For each miRNA, network of miRNA–target interactions (disease-context), based on the experimentally supported miRNA-target data from miRTarBase (https://mirtarbase.cuhk.edu.cn/), is provided using the Human microRNA Disease Database version 4.0 (HMDD v.4.0) (http://www.cuilab.cn/hmdd). Upregulated target genes of miRNAs are in blue color font and downregulated target genes of miRNAs are in black color font.

† For those miRNAs that have not been entered into HMDD v.4.0, predicted targets are indicated according to the miRDB database (https://mirdb.org/).

§ Due to space limit, number of the predicted targets is indicated. Full descriptions of the predicted targets, including target detail, target rank, target score, gene symbol, and gene description, are available at: https://mirdb.org/.
