# Supplementary material for: The Untapped Biomarker Potential of MicroRNAs for Health Risk–Benefit Analysis of Vaping vs. Smoking
Source: Cells. 2024 Aug 10;13(16):1330. doi: 10.3390/cells13161330 (PMC11352591; doi:10.3390/cells13161330)
Supplement: Supplementary file 1 [file cells-13-01330-s001.zip › Table S3.pdf]

Supplementary Table S3. List of differentially expressed miRNAs in plasma exosomes of dual smokers (both cigarettes and waterpipe, *N* = 7) as compared to non-users (*N* = 8) from the Singh *et al*. study (ref. [110]).

**miRNA**	**Log2 fold change**	**t-test *p*-value**	**FDR adjusted *p*-value**	**miR-Target Network ***
hsa-miR-362-5p	-44.12 ↓	2.76E-22	1.30E-19	*CASP8, FGF9*
hsa-miR-29b-3p	-21.55 ↓	8.99E-14	2.12E-11	*FBN1, REST, LOX, MDM2, TET2, CNBP, SMARCC1, LAMA2, COL5A1, BCL2, TNFAIP3, DNMT3A, TGFB2, VHL, IFNG, ESR1, BACE1, CCNA2, NOTCH2, HDAC4, AQP4*
hsa-miR-149-5p	29.29 ↑	1.19E-10	1.87E-08	*TP53, FOSL2, FPGS, LDLR, MAP2K7, CNBP, CALD1, FASLG, HECTD4, AKT1, CDKN1B, HLA-A, TRAF6, AHR, BCL2L1, SLC7A5, CD40LG, BIRC5, IGF1, ITGB3, CDKN1A, ADIPOQ, MLX, TES, YARS2, CALR, MTHFR, IKZF3, IL6, TRIM44, GPRC5A, MYH9, SNRNP200, FGFR1, KIF1A, AIP, OGG1, MYD88*
hsa-let-7i-5p	1.36 ↑	9.16E-07	0.000108	*MDM4, MYBPC3, SOD2, EPHA4, CCND1, EDN1, IKZF3, ACTA1, IGF1, IGF1R, MAP2K7, CRX, IL13*
hsa-miR-21-5p	1.14 ↑	1.47E-05	0.00139	*LATS1, DICER1, MIB1, PTPN14, REST, SLC17A5, RPS6KA3, GDF5, NR2C2, IGF1R, TGFBR2, CYCS, STAT3, RB1, COL4A1, PTPN3, OXTR, SOX11, CCL1, CADM1, LAMP2, DMD, CLCN5, BAZ1B, SLC9A6, GGCX, BCL2, TOP2A, KAT6A, KLF9, MDM4, PTGFR, SLC31A1, ZBTB20, FMR1, FUT2, SEMA5A, CCNG1, HS3ST3B1, PURA, KIF6, CCND1, PPARA, NBEA, CDK6, LIFR, TCF21, WNT5A, FKBP5, SOX5, RECK, PLAT, TRIM44, EIF2S1, TLR4, PPM1L, GTF2I, CEP152, AGAP1, NTF3, FOXO3, HPGD, CPM, HMGB1, EGFR, PIK3R1, GNE, RP2, NIPBL, TIMP3, SOX2, BMI1, MUC1, PREPL*
hsa-miR-144-3p	-2.37 ↓	1.78E-05	0.001397	*ZBTB20, SOD2, ZNF480, SMAD4, PTEN, FGF2, MAP3K8*
hsa-miR-143-3p	1.28 ↑	4.17E-05	0.0024	*PAPPA, ADCY2, STAR, MDM2, NR2C2, MMP14, CNBP, TRAF3IP2, MMP2, AKT1, IDS, COL5A1, MMP9, THRA, GLUL, TNF, IL2RA, IRF1, MAPK1, DNMT3A, KRAS, IKZF3, XIAP, ITGB1, PTPN2, TEP1, PTGS2, PIK3R1, SMAD3, SMYD4, FHIT, LIMK1, IGF1R*
hsa-miR-30a-5p	1.42 ↑	4.58E-05	0.0024	*DGKH, DROSHA, TP53, CTNNB1, PPARD, SOD2, PRKAR1A, FBXO45, SLC38A2, ELOVL5, MAPK8, NPTN, MET, LDLR, OPHN1, HSPA5, ESR2, SLC1A2, PPARGC1B, FOXG1, BCL11A, CREM, CASP3, MPDU1, SH3PXD2A, GNAL, MECP2, MTR, PEX11B, SLC7A5, YWHAE, EEF2, ITGB3, CNP, THBS1, MAPK1, NUFIP2, NCAM1, PDCD10, KRAS, ATRX, CDK6, LIFR, WNT5A, ENTPD4, HDAC1, SCML2, PNPO, KCNN3, TGM2, KPNA1, KMT2A, SP4, EGFR, NDE1, PBRM1, KREMEN1, PPP3R1, IGF1R, RUNX2, PREPL*

hsa-miR-30c-5p	1.69 ↑	4.23E-05	0.0024	*TP53, SERPINE1, PPARGC1B, CTGF, NOTCH1, SUZ12, LIFR, MCL1, SLC7A5, LDLR*
hsa-let-7f-5p	1.05 ↑	7.02E-05	0.003312	*CCNG1, HDAC2, EPHA4, SMARCC1, ATXN2, EDN1, IKZF3, CYP19A1, BAZ1B, IL6, GLUL, CRX, IL13*
hsa-miR-451a †	-2.07 ↓	0.000114	0.004893	*OSR1, CUX2, PSMB8, CXCL16, TARP, ST8SIA4, CDKN2D, MIF, FBLN5, CERK, SAMD4B, CAB39, VAPA, LETM2, MEX3C, USP46, CMTM6, TBC1D9B, PMM2, KIAA1217, MAU2, RNF217, MEGF6, S1PR2, EVL, FBXO33, ATF2, CDKN2B, UCK1, CAV1, C16orf72, DCAF5, RAB5A, CACHD1, LUZP2, EIF2AK3, AKTIP, FAM171A1, TTN, NEDD9*
hsa-miR-10b-5p	-1.66 ↓	0.000212	0.0077	*TPM4, CDKN1A, SREBF1, CREB1, TPM1, CDKN2A, PTEN, NF1, NOTCH2, NR2C2, XIAP, CLDN1, IGF1R, HLA-B*
hsa-let-7a-5p	1.18 ↑	0.000212	0.0077	*MYC, ARG2, MDM2, SIK1, F2R, CRX, IFNLR1, CASP3, EDN1, IGF2, BCL2, AP1S1, MDM4, NPC1, THBS1, CDKN1A, DUSP6, CCNG1, TES, KRAS, CDK6, FXN, IKZF3, BTG1, EPHA4, VCL, MPL, ACTA1*
hsa-miR-424-3p	16.02 ↑	0.000284	0.009574	*RBPJ, TFAP2A, FGFR1, CUL2, HIF1A, TXNIP, YAP1, CCND1, ITGA2, FGF2, CCNE2, MYB, PDE4D*
hsa-miR-139-5p	16.41 ↑	0.000349	0.010992	*PIK3CA, WNT1, DDX6, NOTCH1, HLA-A, FOS, PDGFRA, SMARCA4, HRAS, ZBTB7A, MCL1, STAMBP, BCL2, MET, RBM8A, PNPO*
hsa-miR-100-5p	1.09 ↑	0.000592	0.017454	*AKT1, FKBP5, RB1, IGF1R*
hsa-miR-25-3p	-0.64 ↓	0.000783	0.021753	*CDKN1C, NFIX, TP53, MDM4, ERBB2, NF2, SH2B3, 14-Sep, TNFSF10, FGF2, XPC, CDH1, MDM2, NOTCH2, RECK, DHFR, NRAS, TNFRSF10A*
hsa-let-7g-5p	0.96 ↑	0.00104	0.027277	*NFIX, SOD2, MBD2, MAP2K7, PDLIM5, CRX, CASP3, DISC1, NDUFS1, HMGA1, BCL2L1, IL13, MDM4, IL6R, THBS1, FYN, CCND1, KRAS, IKZF3, MAP3K1, ARID1A, EPHA4, HMGB1, OLR1, KREMEN1, RHD*
hsa-miR-23a-3p	0.72 ↑	0.00192	0.047694	*TSC1, EN2, PPARGC1A, ADAM28, SMAD5, SOD2, LMAN2L, PTEN, SDHD, CHD4, PTPN11, TRPM7, ABCD1, ALDH5A1, SKI, PSAP, STAT3, STS, CXCL12, LDHA, CLDN16, NAV2, FAS, TNFAIP3, CCL8, IL6R, IRF1, GJA1, TSNAX, NUFIP2, RGS5, AMBRA1, KLF12, TGFB2, FKBP5, IKZF3, SLC1A5, MC2R, RFC2, CXCL8, ADK, LPAR1, MOG, C9orf3, NLGN4X, TBL2, SMAD3, MEF2C, PEX26*
hsa-miR-192-5p	-1.21 ↓	0.0021	0.049555	*HCN4, SH2B3, PRKAR1A, MDM2, BARD1, NTRK3, NOD2, FGF2, RB1, CYP24A1, BCL2, PGM3, SLC19A2, TPM4, SCN5A, SPARC, KIF5B, TCF7, EIF2S1, ESR1, CCNY, SIRT1, CAV1, H3F3A, EMX2, DHFR*

Data are derived from ref. [110]. Arrows indicate upregulated (↑) miRNAs and downregulated (↓) miRNAs. FDR = False discovery rate

* For each miRNA, network of miRNA–target interactions (disease-context), based on the experimentally supported miRNA-target data from miRTarBase (https://mirtarbase.cuhk.edu.cn/), is provided using the

Human microRNA Disease Database version 4.0 (HMDD v.4.0) (http://www.cuilab.cn/hmdd). Upregulated target genes of miRNAs are in blue color font and downregulated target genes of miRNAs are in black color font.

† For those miRNAs that have not been entered into HMDD v.4.0, predicted targets are indicated according to the miRDB database (https://mirdb.org/).
